# Lansoprazole associated with a relatively lower gout risk among PPI users: a nationwide retrospective study

**DOI:** 10.1007/s10067-025-07502-z

**Published:** 2025-06-03

**Authors:** Hung-Kai Shih, Yu-Te Su, Sy-Jou Chen, Chen-Chih Chu, Yu‐Ching Chou, Tsung‐Kun Lin, Ching-Kuo Huang, Wen‐Tung Wu, Ming-Hsun Lin

**Affiliations:** 1 Department of Emergency Medicine, Tri-Service General Hospital, National Defense Medical Center, Taipei, Taiwan; 2 Division of Rheumatology/Immunology and Allergy, Department of Internal Medicine, Tri-Service General Hospital, National Defense Medical Center, Taipei 114, Taiwan; 3 School of Public Health, National Defense Medical Center, Taipei City, Taiwan; 4 Department of Pharmacy, Tri-Service General Hospital, National Defense Medical Center, 3 F. No. 38-2, Section 3, Tingzhou Rd., Zhongzheng Dist., Taipei City 100, Taiwan R.O.C.; 5 School of Pharmacy, National Defense Medical Center, Taipei 114, Taiwan; 6 Department of Pharmacy, Tri-Service General Hospital Songshan Branch, Taipei, Taiwan; 7 Division of Endocrinology and Metabolism, Department of Internal Medicine, Tri-Service General Hospital, National Defense Medical Center, No. 325, Section 2, Cheng-Kung Road, Neihu Dist., Taipei 114, Taiwan R.O.C.

**Keywords:** Gout, Lansoprazole, National Health Insurance Research Database (NHIRD), Proton pump inhibitor (PPI)

## Abstract

**Introduction:**

Proton pump inhibitors (PPIs) used for gastrointestinal-related disorders are associated with increased insulin resistance, a risk factor for worsening hyperuricemia. However, lansoprazole has shown potential in reducing insulin resistance by increasing the expression of peroxisome proliferator-activated receptor gamma and CCAAT/enhancer-binding protein alpha mRNA in adipogenesis. This study aims to investigate the effects of lansoprazole compared to other PPIs in reducing the risk of gout.

**Method:**

We conducted a retrospective cohort study on patients using lansoprazole from 2000 to 2005, based on the Taiwan National Health Insurance Database, with follow-up until 2013. A comparison cohort on other PPIs was selected through propensity score matching for age, sex, comorbidities, and concomitant medications. Gout risk was analyzed using survival analysis and a Cox proportional hazards model.

**Results:**

Among 1816 lansoprazole users, 139 developed gout (7.7%), compared to 968 (13.3%) out of 7264 in the other PPIs group. The average age was 53.33 (± 14.79) in the lansoprazole group and 52.71 (± 14.81) in the other PPIs group. The cumulative incidence of gout was lower in the lansoprazole cohort, with a significantly reduced gout risk (adjusted hazard ratio, 0.64; 95% CI, 0.56–0.73). This lower risk remained after stratification by gender and among individuals over 30 years old.

**Conclusions:**

Among PPI users, lansoprazole is associated with a significantly lower risk of gout. For patients at risk of gout who require PPI therapy, lansoprazole may be considered the treatment of choice.

**Supplementary Information:**

The online version contains supplementary material available at 10.1007/s10067-025-07502-z.

**Table:** 

**Key Points** • *The cumulative incidence of gout is lower in lansoprazole users than in other PPI users* • *Lansoprazole users had a significant lower risk of developing gout compared to other PPI users* • *Lansoprazole may be considered for patients at risk of gout*	

## Introduction

Proton pump inhibitors (PPIs) are one of the most frequently prescribed drug classes, with approximately one-quarter of adults using them globally [1]. The most common indications for PPIs were gastroprotection for gastrointestinal irritant medications, followed by gastroesophageal reflux disease (GERD), gastritis, and gastrointestinal bleeding. While generally considered safe, long-term PPI use has been associated with adverse effects such as gastrointestinal infections, mineral and vitamin malabsorption, and chronic kidney disease. Additionally, PPI use also links to unexpected acute and chronic conditions, such as stroke and dementia [2, 3]. In parallel, recent studies have increasingly explored how bioactive compounds may modulate systemic inflammation and metabolic regulation [4, 5], reflecting a broader scientific interest in agents including pharmaceuticals that influence chronic disease pathways through diverse mechanisms. A recent population-based cohort study demonstrated that PPI use was associated with increased insulin resistance as measured by HOMA-IR levels; possible mechanisms include PPI-induced hypomagnesemia and suppression of insulin-like growth factor-1(IGF-1) due to PPIs [6–9]. Also, a systemic review on 2024 identified 12 studies (8 cohort, 1 RCT, and 3 case–control) with a total of 12, 64, and 816 population concludes that chronic PPI exposure increases the risk of T2DM incidence [10]. Since insulin resistance is a recognized contributor to hyperuricemia and impaired uric acid excretion, these findings suggest a mechanistic link between PPI exposure and gout risk. Notably, a case–control population study demonstrated that the use of PPIs increases the risk of developing gout by 30% [11]. Furthermore, a pharmacokinetic study on volunteers found that a single dose of ranitidine or omeprazole led to a significant increase in blood uric acid levels within 12 h [12]. These results indicate that PPIs may increase uric acid levels and the risk of developing gout.

Gout is a widespread health issue caused by a disruption in purine metabolism, leading to high uric acid levels and urate crystal buildup in joints and tissues. Emerging evidence strongly links gout with metabolic syndrome, including diabetes, hypertension, and hyperlipidemia [13]. Moreover, hyperuricemia and insulin resistance may have a bidirectional causal relationship [14]. Insulin resistance can reduce the kidney’s ability to excrete uric acid by upregulating urate transporter-1 (URAT1) in renal tubular epithelial cells, thereby increasing serum urate concentrations and contributing to gout pathogenesis [15]. In addition to this renal mechanism, insulin resistance is a hallmark of metabolic syndrome, which shares overlapping features with gout, such as systemic inflammation, endothelial dysfunction, and oxidative stress. An animal study found that urate transporter-1 (URAT1), a transporter responsible for uric acid reabsorption in the kidneys, was also found in adipose tissue. Selective inhibitors of URAT1, used to treat hyperuricemia, can improve insulin resistance induced by a high-fat diet [16]. This highlights insulin resistance as a central pathophysiologic factor in gout development. Given the persistent global impact of metabolic syndrome, lowering uric acid levels may offer an effective strategy for controlling this condition.

Lansoprazole, one of the PPIs, has been found to decrease insulin resistance though adipocyte regulation. An animal study found that lansoprazole promotes adipogenesis by upregulating key transcription factors, such as peroxisome proliferator-activated receptor gamma (PPARγ) and CCAAT/enhancer-binding protein alpha (C/EBPα), which improve glucose uptake and insulin sensitivity at pharmacological doses [17]. At higher concentrations, lansoprazole inhibits adipocyte differentiation by downregulating these factors, which reduces fat mass and glucose uptake. Despite lowering insulin sensitivity in the short term, the overall reduction in fat mass and body weight could improve metabolic health and reduce obesity-related complications, suggesting its potential benefits for metabolic syndrome [17, 18]. These studies indicate that lansoprazole may promote adipogenesis and reduce insulin resistance, which help improve hyperuricemia. Considering the positive effects of lansoprazole on insulin resistance compared to other PPIs, we hypothesize that lansoprazole is associated with a relatively lower gout risk among PPI users.

## Materials and methods

### Data source

This retrospective cohort study utilized the National Health Insurance Research Database (NHIRD) in Taiwan. Established in 1995, the National Health Insurance (NHI) program is a publicly funded single-payer health insurance initiative providing coverage to all residents of Taiwan. The NHIRD contains comprehensive medical information, encompassing demographic data, clinical visits, diagnostic codes following the International Classification of Diseases, Ninth Revision, Clinical Modification (ICD-9-CM), and prescription details. Recognized for its robustness, the NHIRD serves as a foundation for high-quality epidemiological studies, offering reliable information on diagnoses and drug prescriptions [19–21].

The study data were derived from the Longitudinal Health Insurance Database 2000 (LHID 2000), a subset of the NHIRD. The LHID 2000 dataset comprises historical ambulatory and inpatient care information for one million randomly sampled beneficiaries enrolled in the NHI system in 2000. This database facilitates an in-depth exploration of the medical service utilization history of the selected patients. Notably, no significant variations in age, sex, or healthcare costs were observed between individuals in LHID and NHIRD [22].

### Ethical considerations

Since the data set was released for research purposes and contained only de-identified information on patient identification, informed consent from the subjects was deemed exempt. Furthermore, the study protocol received approval from the Institutional Review Board of Tri-Service General Hospital (TSGHIRB No. A202405037).

### Study subjects

#### Population selection

**Fig. 1:**
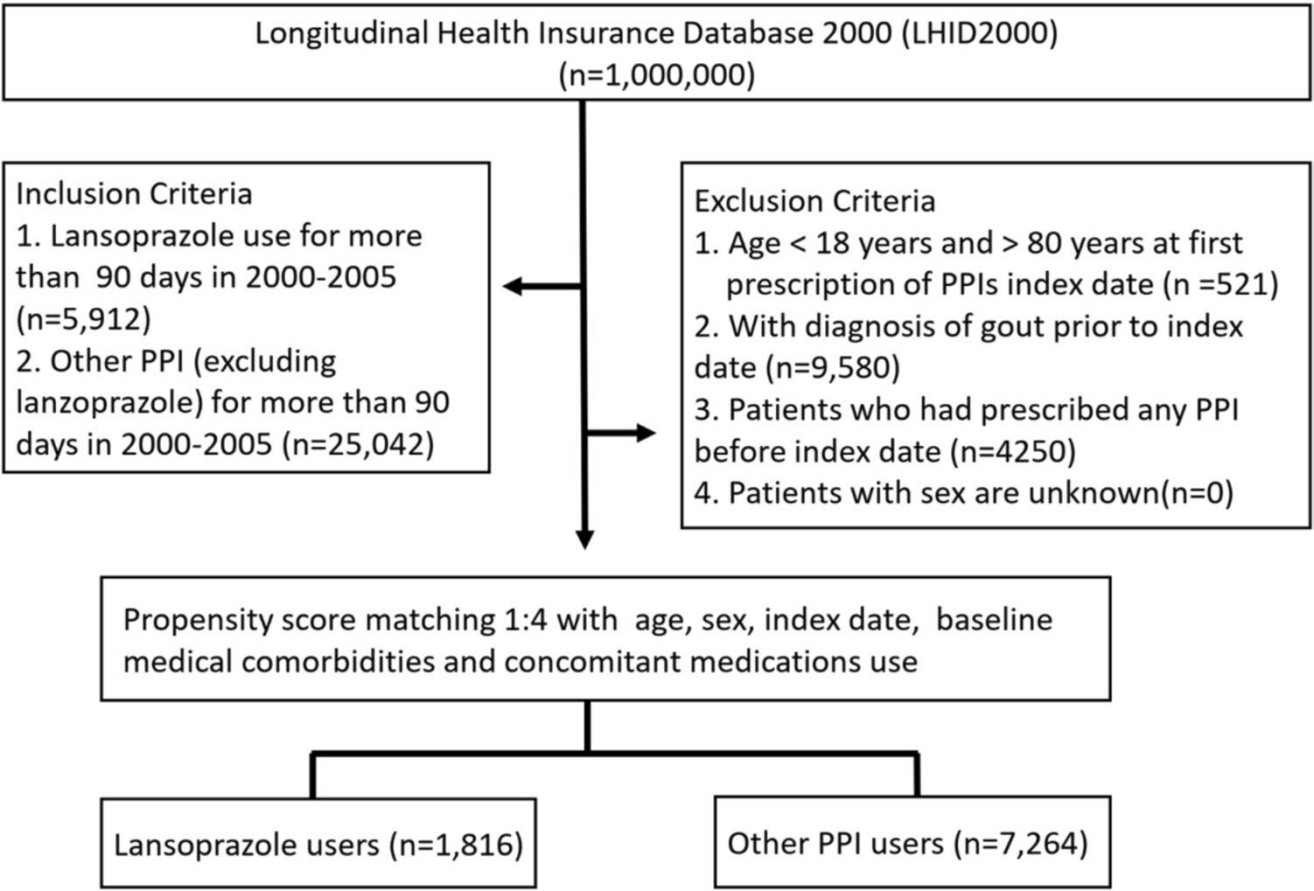
Flow chart of the study design

The study cohort was drawn from patients randomly selected into the Longitudinal Health Insurance Database 2000 (LHID 2000) between January 1, 2000, and December 31, 2005. The inclusion criteria specified individuals aged between 20 and 80 years, ensuring continuous coverage by the National Health Insurance (NHI) during the specified period. Exclusion criteria for both the lansoprazole and comparison PPI groups were as follows (as Fig. 1): (1) patients aged < 18 or > 80 years; (2) patients diagnosed with gout (ICD-9-CM: 274) or hyperuricemia (ICD-9-CM: 790.6) prior to the index date; (3) patients who had used any PPI before January 1, 2000; and (4) patients who received overlapping prescriptions for both lansoprazole and other PPIs during the exposure period. These criteria were applied to reduce confounding by prior medication use or underlying urate-related conditions and to ensure mutually exclusive exposure groups for analysis.

#### Exposure definition

Exposure to lansoprazole (Anatomic Therapeutic Chemical (ATC) code: A02BC03) and other proton pump inhibitors (PPIs), including omeprazole (ATC code: A02BC01), esomeprazole (ATC code: A02BC05), pantoprazole (ATC code: A02BC02), and rabeprazole (ATC code: A02BC04), was collected between January 1, 2000, and December 31, 2005. The exposed cohort consisted of patients receiving lansoprazole without concurrent prescriptions for other PPIs during the exposure period, while the comparison cohort comprised patients receiving other PPIs without lansoprazole use during the study period. We used cumulative defined daily dose (cDDD), computed as the sum of dispensed DDDs of PPIs within the 5 years and enrolled subjects with cDDD > 90. Before the index date, patients in both cohorts had no prior diagnosis of gout (ICD 274) or hyperuricemia (ICD 790.6).

#### Covariate assessment and adjustment

Covariates for the study included patients’ demographic characteristics, baseline comorbidities, and the utilization of co-medications. Demographic information and the presence of comorbidities were assessed through outpatient files, with the latter considered present if a patient received a diagnosis for any of the specified diseases in at least two outpatient claims during the study period. Additionally, information on concomitant medication usage was retrieved from NHIRD prescription database using the ATC classification system codes.

#### Propensity score matching and final cohort

To mitigate potential confounders between cohorts, propensity score matching was employed at a ratio of 1:4 for the exposed and comparison cohorts. After applying these criteria, the study included 1816 patients in the exposed cohort and 7264 patients in the comparison cohort (Fig. 1). Propensity scores were calculated for each patient using a logistic regression model incorporating covariates such as age, sex, index date, comorbidities, including cerebral vascular disease (ICD-9-CM codes: 430–438), chronic liver disease (ICD-9-CM codes: 570–572), chronic kidney disease (ICD-9-CM code: 585), hyperlipidemia (ICD-9-CM code: 272), hypertension (ICD-9-CM codes: 401–405), diabetes mellitus (ICD-9-CM code: 250), malignancy (ICD-9-CM codes: 140–208), alcoholic related illness (ICD-9-CM code: 291, 303, 305, 571.0, 571.1, 571.2, 571.3, 571.5, 571.6), rheumatoid arthritis (ICD-9-CM codes: 714.), coronary artery disease ((ICD-9-CM code: 410–414), heart failure (ICD-9-CM codes: 402–404), and co-medications, including aspirins (ATC code: A01 AD05), thiazide (ATC code: C03 AA), pyrazinamide (ATC code: J04 AK01, J04 AM05, J04 AM06), ethambutol (ATC code: J04 AK02, J04 AM03, J04 AM07), and furosemide (ATC code: C03 CA).

#### Follow-up and outcome assessment

Subjects using PPIs between January 1, 2000, and December 31, 2005, were followed from the index date until the onset of gout. For each subject, follow-up duration in person-years was determined starting from the index date and continued until the earliest occurrence of one of the following events: a new diagnosis of gout, loss to follow-up, death, withdrawal from the national insurance program, or the study endpoint in 2013.

### Statistical analysis

Descriptive statistics were used to characterize the baseline characteristics of the study cohorts. Chi-square and *t*-tests were used to evaluate the distributions of categorical and continuous variables between the study cohorts. Differences in the cumulative risk of gout between the cohorts were estimated using the Kaplan–Meier method, and differences between cohorts were evaluated with the log-rank test. A multivariable Cox regression model, which included relevant covariates and the propensity score as a covariate, was used to estimate the association between lansoprazole use and gout risk. Covariates included age, sex, comorbidities such as diabetes, hypertension, hyperlipidemia, and chronic kidney disease, and the use of medications known to affect uric acid levels, including thiazide diuretics, aspirin, and anti-tuberculosis drugs. To further evaluate potential effect modification and residual confounding, we conducted extensive stratified analyses by baseline comorbidities and medication use. These results, presented in Supplementary Table S1, demonstrate that the association between lansoprazole use and reduced gout risk remained consistent across most subgroups, supporting the robustness of our findings. The results were presented as hazard ratios (HRs) and their 95% confidence intervals (CIs). The proportional hazards assumption was tested using the Schoenfeld residual test, which confirmed that explanatory variables satisfied the proportionality assumption of the Cox regression model (*p* = 0.5318). All statistical tests were two-sided, and a level of 0.05 was considered statistically significant. All data analyses were performed using SAS software, version 9.1 (SAS Institute, Cary, NC).

## Result

### Baseline characteristics of study cohorts

The baseline characteristics of the study cohorts are presented in Table 1. The study involved 1816 participants in the lansoprazole group and 7264 participants in the other PPIs group. The average age of participants in the lansoprazole group was 53.33 years (± 14.79), while the average age in the other PPIs group was 52.71 years (± 14.81) (*p* value 0.114). There were no significant differences in the distributions of other characteristics including sex, comorbidities, and concomitant medications between the lansoprazole cohort and the comparison cohort.

**Table 1:** Baseline characteristics of study cohorts

Variable	Study cohorts		*p* value
	Other PPI (*N* = 7264)	Lansoprazole (*N* = 1816)	
Age (mean ± SD)	52.71 ± 14.81	53.33 ± 14.79	0.114
Gender (no., %)			0.983
Female	3182 (43.8%)	796 (43.8%)	
Male	4082 (56.2%)	1020 (56.2%)	
Comorbidities (no., %)			
Cerebral vascular disease	1529 (21.0%)	368 (20.3%)	0.462
Chronic liver disease	1952 (26.9%)	498 (27.4%)	0.636
Chronic kidney disease	454 (6.2%)	96 (5.3%)	0.124
Hyperlipidemia	1075 (14.8%)	270 (14.9%)	0.941
Hypertension	2857 (39.3%)	713 (39.3%)	0.957
Diabetes mellitus	1938 (26.7%)	491 (27.0%)	0.758
Malignancy	1159 (16.0%)	265 (14.6%)	0.153
Alcoholic related illness	845 (11.6%)	229 (12.6%)	0.249
Rheumatoid arthritis	229 (3.2%)	61 (3.4%)	0.654
Coronary artery disease	1587 (21.8%)	397 (21.9%)	0.99
Congestive heart failure	328 (4.5%)	73 (4.0%)	0.358
Concomitant medications			
Aspirin	2317 (31.9%)	553 (30.5%)	0.236
Thiazide	703 (9.7%)	163 (9.0%)	0.362
Pyrazinamide	158 (2.2%)	30 (1.7%)	0.161
Ethambutol	168 (2.3%)	41 (2.3%)	0.889
Furosemide	1744 (24.0%)	429 (23.6%)	0.731

### The association of lansoprazole with the risk of gout

The association between lansoprazole use and the risk of developing gout are presented in Table 2. Among the participants, 139 out of 1816 in the lansoprazole group developed gout, compared to 968 out of 7264 in the other PPIs group. After adjusting for potential confounding factors, the lansoprazole-user cohort showed a significantly lower incidence of gout compared to the comparison cohort (adjusted hazard ratios (HRs), 0.64; 95% confidence interval (CI), 0.56–0.73). The adjusted HR (95% CI) for males was 0.64 (0.51–0.79), and for females, 0.63 (0.45–0.87), indicating a consistent risk reduction across genders. For age groups of 18–29, 30–59 and 60–79, the adjusted HRs (95% CIs) were 0.83 (0.34–2.06), 0.59 (0.45–0.76), and 0.67 (0.52–0.87), respectively. Overall, the risk of gout remained lower in lansoprazole users after stratification by genders and in individuals of age over 30 s.

**Table 2:** Comparison of hazard ratios of gout stratified by sex and age

Variable	No. of subjects	No. of gout cases	Crude HR (95% CI)	Adjusted HR (95% CI)
Overall				
Other PPI	7264	968	1	1
Lansoprazole	1816	139	0.66 (0.58–0.75)	0.64 (0.56–0.73)
Gender				
Males				
Other PPI	4082	674 (16.5%)	1	1
Lansoprazole	1020	97 (9.5%)	0.65 (0.52–0.80)	0.64 (0.51–0.79)
Females				
Other PPI	3182	294 (9.2%)		
Lansoprazole	796	42 (5.3%)	0.65 (0.47–0.90)	0.63 (0.45–0.87)
Age (years)				
18–29				
Other PPI	488	33 (6.4%)	1	1
Lansoprazole	122	6	0.79 (0.33–1.89)	0.83 (0.34–2.06)
30–59				
Other PPI	4116	497 (10.9%)	1	1
Lansoprazole	1029	65	0.61 (0.47–0.79)	0.59 (0.45–0.76)
60–79				
Other PPI	2660	438 (15.2%)	1	1
Lansoprazole	665	68	0.68 (0.53–0.88)	0.67 (0.52–0.87)

Hazard ratios were adjusted for age, sex, index date, comorbidities, including cerebral vascular disease, chronic liver disease, chronic kidney disease hyperlipidemia, hypertension, diabetes mellitus, malignancy, alcoholic related illness, rheumatoid arthritis, coronary artery disease, and congestive heart failure, and the use of concomitant medications, including beta blocking agents, statins, clopidogrel, and aspirin

*DM* diabetes mellitus, *HR* hazard ratio, *CI* confidence interval

### Comparisons the cumulative incident of gout

**Fig. 2:**
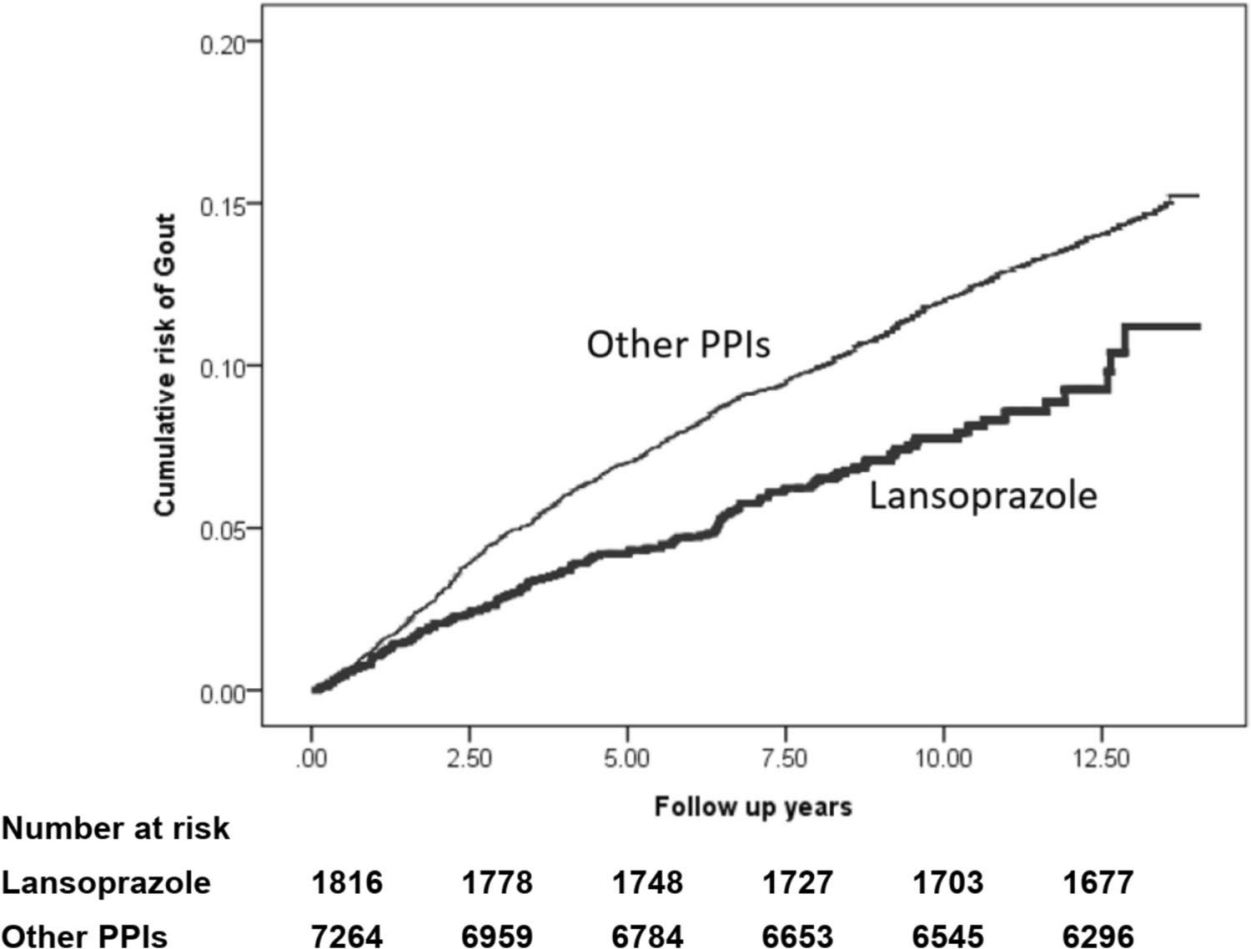
Kaplan–Meier curves for the cumulative incident of gout stratified by administration of lansoprazole or other PPIs

The median follow-up length was 8.80 years in the lansoprazole cohort and 9.86 years in the comparison cohort. The cumulative incidence of gout in lansoprazole users was significantly lower than in other PPIs users (*p* < 0.05), indicating a clinically meaningful reduction in risk over the follow-up period (Fig. 2).

## Discussion

This retrospective cohort study revealed that patients using lansoprazole had a lower risk of developing gout compared to those using other PPIs. This effect was consistently observed across gender and age groups, suggesting that for patients at risk of gout who need PPIs, lansoprazole may be a preferable choice.

Studies have indicated that long-term PPI use may be linked to a higher risk of gout. Several possible explanations have been proposed. First, a prospective population-based cohort found that PPI use increased insulin resistance, as measured by Homeostatic Model Assessment of Insulin Resistance (HOMA-IR) values, may be associated with PPI use [9]. PPI can cause hypomagnesemia and lower insulin growth factor (IGF)−1 levels, which are linked to insulin resistance [6, 7]. Insulin resistance enhances uric acid reabsorption by upregulating the expression of the renal urate transporter URAT1, a protein in the kidney responsible for reabsorbing uric acid from urine back into the bloodstream [16], and is considered an independent risk factor for the development of hyperuricemia and gouty arthritis [23]. Second, antacids inhibit the gastric H +/K + ATPase, reducing ATP turnover, which can lead to excess ATP being metabolized into adenine and ultimately converted into uric acid [12]. Third, PPIs may inhibit the renal H +/K + -ATPase due to its structural similarity to the gastric enzyme. This inhibition can disrupt acid–base balance, leading to metabolic alkalosis. In an alkaline environment, uric acid becomes less soluble, reducing its excretion by the kidneys and potentially causing hyperuricemia [24]. Finally, PPIs have been linked to the development of acute interstitial nephritis, which is associated with chronic kidney disease and reduced uric acid excretion [25].

Lansoprazole is associated with a lower risk of gout compared to other PPIs, potentially due to its unique ability to improve insulin resistance through metabolic modulation. While all PPIs have been associated with adverse metabolic effects—such as reduced serum magnesium and insulin-like growth factor-1 (IGF-1) levels—which may contribute to insulin resistance, impaired renal urate excretion, and systemic inflammation, lansoprazole appears to differ mechanistically. Specifically, lansoprazole has been shown to enhance insulin sensitivity through adipocyte browning, where white fat cells are converted into energy-burning brown-like cells [26, 27], and adipogenesis, the formation of new adipocytes from precursor cells, which enhances lipid storage capacity and reduces the burden on existing adipocytes [28]. These effects are mediated by the upregulation of PPAR-γ, a transcription factor that regulates adipocyte differentiation, glucose homeostasis, and inflammation, ultimately leading to reduced insulin resistance and lower risk of type 2 diabetes [17, 18, 29]. Supporting this mechanism, previous research of pioglitazone, a ligand for PPAR-γ, demonstrated a 19% risk reduction in gout development compared to non-pioglitazone users [30], and diabetic patients using PPAR-γ agonists have exhibited a reduced incidence of gout compared to non-PPAR-γ agonist users. Thus, lansoprazole may counterbalance the class-wide metabolic liabilities of PPIs through its distinct PPAR-γ–related pathway.

Our findings are consistent with a recent population-based case–control study by Zhu et al. (2023), which demonstrated that PPI use is associated with an increased risk of gout in the general population, highlighting the importance of evaluating individual PPIs rather than the class as a whole [11]. Additionally, our earlier cohort study showed that lansoprazole use was associated with a lower risk of type 2 diabetes, supporting its potential metabolic benefits and reduced gout risk compared to other PPIs [29]. These two recent studies contribute to an emerging body of evidence suggesting lansoprazole may have unique metabolic advantages among PPIs. Our findings provide further support for this hypothesis and emphasize the importance of considering PPI selection in clinical decision-making. Although our methodology differs from that of Zhu et al. (2023), variations in study design and analytical approach may contribute to these differences; further large-scale prospective studies or more comprehensive mechanistic research will be necessary to confirm these findings.

Several limitations are present in this retrospective study. First, the NHIRD’s use of encrypted individual identifiers restricts access to important data, including family history of gout, laboratory results like uric acid levels, and imaging findings. It also does not capture individual-level data on ethnic background or socioeconomic status, which may influence gout risk or healthcare access. This limits our ability to assess these potentially confounding variables and may affect the generalizability of the findings to more diverse populations. Second, despite adjustments for age, sex, comorbidities, and other medications, some confounding factors might still exist. In particular, the database does not include lifestyle-related variables such as dietary habits, alcohol consumption, or physical activity, which may influence the risk of hyperuricemia and gout. Due to the inherent nature of being a retrospective study, it is not possible to directly verify actual insulin resistance or other mechanisms. Future studies should explore the underlying mechanisms and confirm these findings in randomized controlled trials. This could offer a better therapeutic option for PPI users at risk of gout. Additionally, research is needed to examine the effects of each type of PPI on gout.

## Conclusion

Our study found that lansoprazole is associated with a significantly lower risk of developing gout compared to other PPIs. This effect was observed across various demographics, including both genders and in age groups over 30 s. Lansoprazole may be considered a preferable choice for patients at risk of gout.

## Supplementary Information

Below is the link to the electronic supplementary material.

Supplementary file1 (DOCX 22 KB)

## Data Availability

The data sets used in the present study are not available based on the policy of using nationwide insurance claims datasets by the Ministry of Health and Welfare in Taiwan. Correspondence and requests for materials should be addressed to Wen‐Tung Wu or Ming-Hsun Lin.

## Abbreviations

*PPI*: Proton pump inhibitor
*NHIRD*: National Health Insurance Research Database
*PPAR*γ: Peroxisome proliferator-activated receptor gamma
*C/EBP*α: CCAAT/enhancer-binding protein alpha
*HR*: Hazard ratio
*CI*: Confidence interval
*cDDD*: Cumulative defined daily dose
*ATC*: Anatomical Therapeutic Chemical Classification System
*ICD-9-CM*: International Classification of Diseases, Ninth Revision, Clinical Modification
*URAT1*: Urate transporter-1
