# Supplementary material for: Lansoprazole associated with a relatively lower gout risk among PPI users: a nationwide retrospective study
Source: Clin Rheumatol. 2025 Jun 3;44(7):2971–9. doi: 10.1007/s10067-025-07502-z (PMC12234581; doi:10.1007/s10067-025-07502-z)
Supplement: Supplementary file 1 — Supplementary file1 (DOCX 22 KB) [file 10067_2025_7502_MOESM1_ESM.docx]

| Table S1. Subgroup analysis of the association between administration of LANSOPRAZOLE, other PPI and risk of gout on the basis of baseline comorbidities | | | | |
| --- | --- | --- | --- | --- |
| Stratified variable | Crude HR  (95% CI) | p-value | Adjusted HR (95% CI) | p-value |
| Baseline comorbidities | |  |  |  |
| Cerebral vascular disease |  |  |  |  |
| Without | 0.59 (0.48 - 0.72) | <0.001 | 0.58 (0.47 - 0.71) | <0.001 |
| With | 0.95 (0.65 - 1.38) | 0.792 | 0.93 (0.64 - 1.35) | 0.693 |
| Chronic liver disease |  |  |  |  |
| Without | 0.61 (0.50 - 0.75) | <0.001 | 0.59 (0.48 - 0.73) | <0.001 |
| With | 0.78 (0.55 - 1.09) | 0.146 | 0.77 (0.54 - 1.08) | 0.125 |
| Chronic kidney disease, |  |  |  |  |
| Without | 0.62 (0.52 - 0.75) | <0.001 | 0.62 (0.51 - 0.74) | <0.001 |
| With | 1.45 (0.70 - 3.03) | 0.317 | 1.44 (0.68 - 3.07) | 0.345 |
| Hyperlipidemia |  |  |  |  |
| Without | 0.60 (0.49 - 0.73) | <0.001 | 0.58 (0.47 - 0.71) | <0.001 |
| With | 0.88 (0.61 - 1.28) | 0.515 | 0.88 (0.61 - 1.27) | 0.49 |
| Hypertension |  |  |  |  |
| Without | 0.53 (0.40 - 0.70) | <0.001 | 0.51 (0.39 - 0.67) | <0.001 |
| With | 0.77 (0.61 - 0.98) | 0.03 | 0.76 (0.60 - 0.96) | 0.023 |
| Diabetes mellitus |  |  |  |  |
| Without | 0.60 (0.48 - 0.74) | <0.001 | 0.59 (0.47 - 0.73) | <0.001 |
| With | 0.79 (0.58 - 1.09) | 0.149 | 0.78 (0.56 - 1.07) | 0.121 |
| Malignancy |  |  |  |  |
| Without | 0.59 (0.49 - 0.72) | <0.001 | 0.59 (0.49 - 0.72) | <0.001 |
| With | 1.48 (0.85 - 2.58) | 0.165 | 1.49 (0.85 - 2.60) | 0.164 |
| Alcoholic related illness |  |  |  |  |
| Without | 0.65 (0.54 - 0.79) | <0.001 | 0.64 (0.53 - 0.77) | <0.001 |
| With | 0.61 (0.32 - 1.19) | 0.146 | 0.66 (0.34 - 1.29) | 0.226 |
| Rheumatoid arthritis |  |  |  |  |
| Without | 0.64 (0.53 - 0.77) | <0.001 | 0.63 (0.52 - 0.75) | <0.001 |
| With | 0.93 (0.38 - 2.24) | 0.864 | 0.90 (0.35 - 2.32) | 0.83 |
| Coronary artery disease |  |  |  |  |
| Without | 0.60 (0.49 - 0.74) | <0.001 | 0.59 (0.48 - 0.73) | <0.001 |
| With | 0.82 (0.58 - 1.15) | 0.248 | 0.79 (0.56 - 1.11) | 0.168 |
|  |  |  |  |  |
| Congestive heart failure |  |  |  |  |
| Without | 0.65 (0.54 - 0.78) | <0.001 | 0.63 (0.53 - 0.76) | <0.001 |
| With | 0.64 (0.27 - 1.49) | 0.298 | 0.69 (0.29 - 1.64) | 0.403 |
|  |  |  |  |  |
| Concomitant medications |  |  |  |  |
| Aspirin |  |  |  |  |
| Without | 0.54 (0.43 - 0.69) | <0.001 | 0.52 (0.41 - 0.66) | <0.001 |
| With | 0.87 (0.66 - 1.14) | 0.308 | 0.85 (0.65 - 1.12) | 0.248 |
| Thiazide |  |  |  |  |
| Without | 0.63 (0.52 - 0.76) | <0.001 | 0.61 (0.51 - 0.74) | <0.001 |
| With | 0.86 (0.52 - 1.42) | 0.56 | 0.83 (0.50 - 1.38) | 0.474 |
| Pyrazinamide |  |  |  |  |
| Without | 0.65 (0.55 - 0.78) | <0.001 | 0.64 (0.53 - 0.76) | <0.001 |
| With | 0.50 (0.12 - 2.11) | 0.345 | 0.34 (0.06 - 1.90) | 0.218 |
| Ethambutol |  |  |  |  |
| Without | 0.65 (0.54 - 0.78) | <0.001 | 0.63 (0.53 - 0.76) | <0.001 |
| With | 0.57 (0.17 - 1.90) | 0.362 | 0.52 (0.14 - 1.96) | 0.334 |
| Furosemide |  |  |  |  |
| Without | 0.58 (0.47 - 0.72) | <0.001 | 0.57 (0.46 - 0.71) | <0.001 |
| With | 0.89 (0.64 - 1.25) | 0.501 | 0.88 (0.63 - 1.24) | 0.468 |

|  |  |  |  |  |
| --- | --- | --- | --- | --- |

HR, hazard ratios, CI, confidence interval.

Hazard ratios were adjusted for age, sex, index data, Cerebral vascular disease, Chronic liver disease, Chronic kidney disease, Hyperlipidemia, Hypertension, Diabetes mellitus, Malignancy, Alcoholic related illness, Rheumatoid arthritis, Coronary artery disease, Congestive heart failure as well as use of concomitant medications, including beta blocking agents, statins, clopidogrel and aspirin
